# Supplementary material for: Multimodal single-cell profiling reveals neuronal vulnerability and pathological cell states in focal cortical dysplasia
Source: iScience. 2024 Nov 6;27(12):111337. doi: 10.1016/j.isci.2024.111337 (PMC11617397; doi:10.1016/j.isci.2024.111337)
Supplement: Document S1. Figures S1–S4 [file mmc1.pdf]

iScience, Volume *27*

Supplemental information

Multimodal single-cell profiling reveals neuronal vulnerability and pathological cell states in focal cortical dysplasia

Isabella C. Galvão, Manuela Lemoine, Lauana A. Messias, Patrícia A.O.R.A. Araújo, Jaqueline C. Geraldis, Clarissa L. Yasuda, Marina K.M. Alvim, Enrico Ghizoni, Helder Tedeschi, Fernando Cendes, Fabio Rogerio, Iscia Lopes-Cendes, and Diogo F.T. Veiga

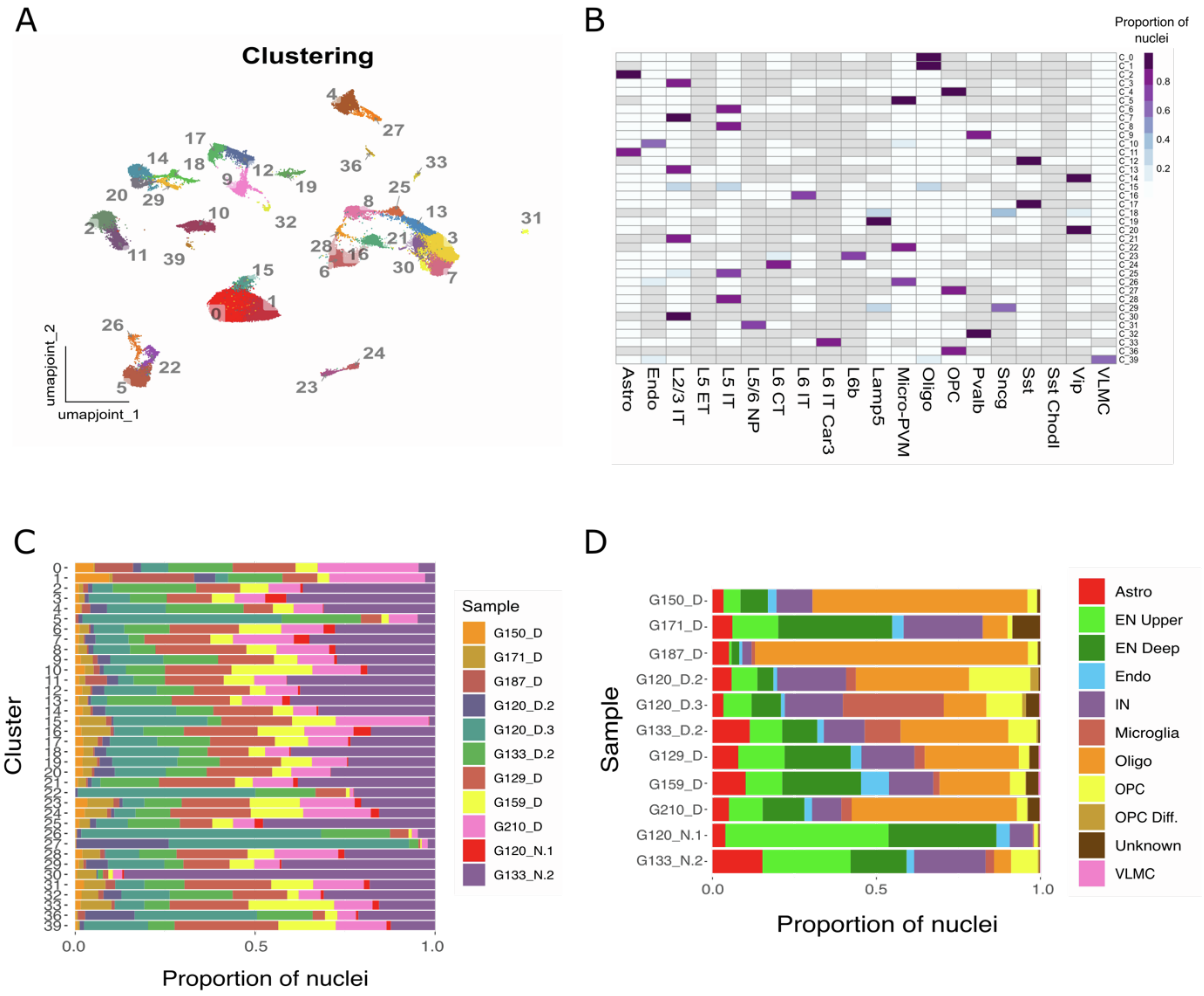

Figure S1, related to Figure 1. Clustering and annotation.

(A) UMAP visualization of nuclei clusters in the multimodal FCD dataset. Clustering was performed in the joint graph (ATAC + RNA) obtained by Weighted Nearest Neighbor (WNN) analysis.

(B) Heatmap of cluster annotation performed by Azimuth based on the Allen atlas of the human cortex. The color indicates the proportion of Azimuth subtypes identified in each cluster.

(C) Bar graphs denoting the distribution of samples in each cluster.

(D) Bar graphs showing the distribution of final consensus cell types across nuclei clusters.

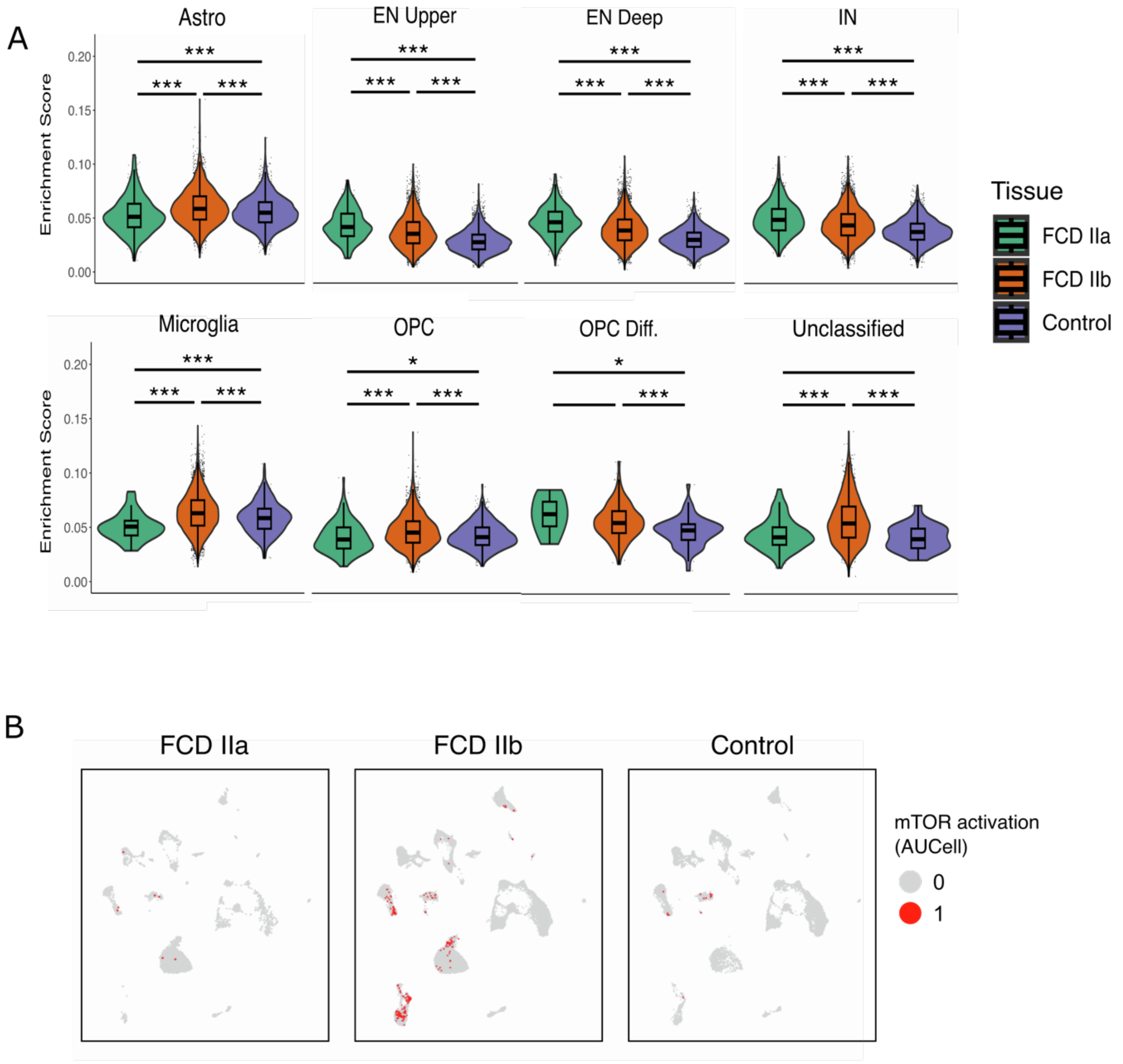

Figure S2, related to Figure 1. Expression of mTORC1 signaling across cell types in FCD lesions.

(A) Violin plots of the mTORC1 signaling enrichment score by cell type and tissue condition. The enrichment score was calculated using the AddModuleScore function from Seurat, using the mTORC1 signature from MSigDB (https://www.gsea-msigdb.org/gsea/msigdb). In box plots, the median is indicated by the center line; box limits represent upper and lower quartiles; and whiskers extend to 1.5 times the interquartile range. Significant changes in cell types were detected using a Wilcox ran-sum test. *P < 0.5, **P < 0.01, ***P < 0.001.

(B) Feature plots showing cells identified as active for the mTORC1 signature by the AUCell method. Cells with scores higher and lower than the AUCell threshold are shown in red and gray, respectively.

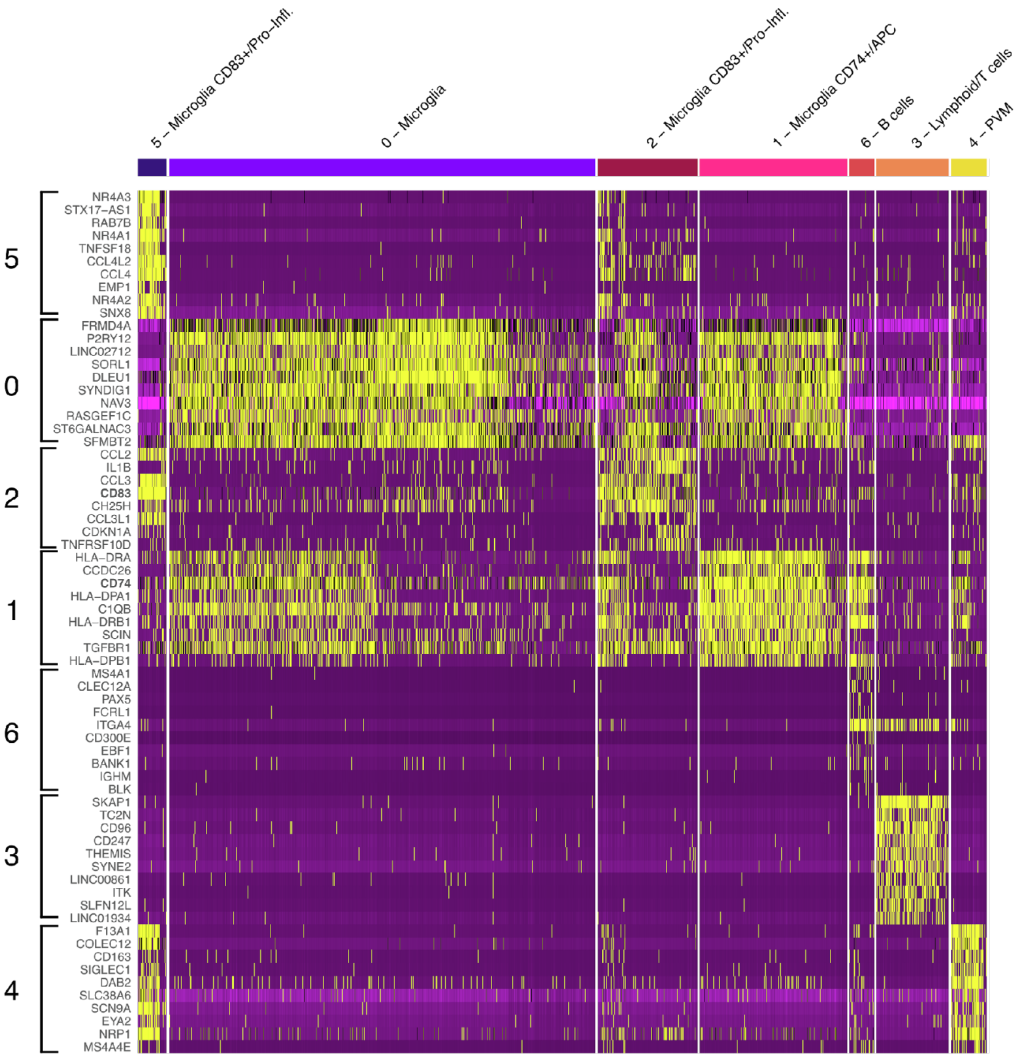

Figure S3, related to Figure 4 and Table S1. Heatmap depicting expression of top 10 markers in microglia subclusters. Markers were computed using the function FindMarkers from Seurat using the Wilcox test, with a min.pct of 0.1, adjusted $P < 0.05$, and log2 fold-change > 1.5.

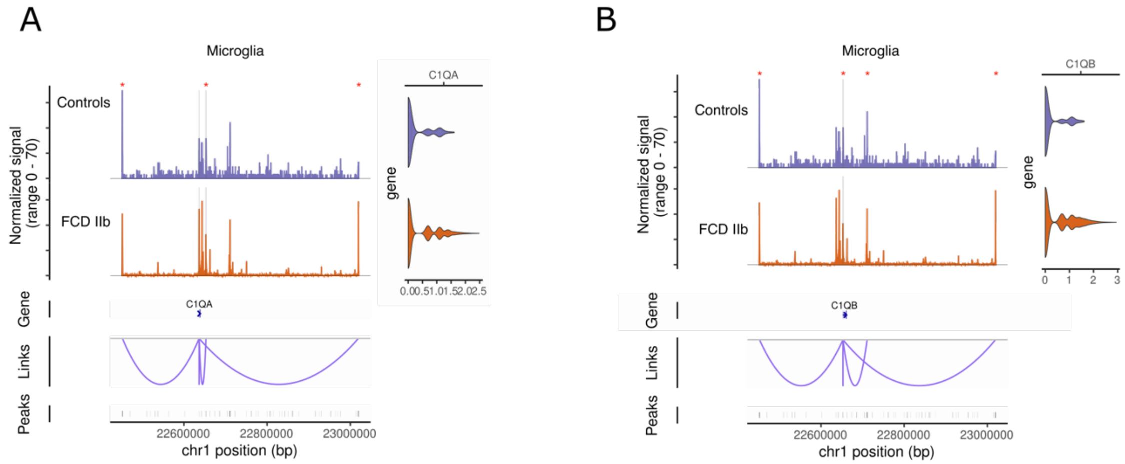

Figure S4, related to Figure 5. Open chromatin regions linked to *C1QA* and *C1QB* regulation FCD IIb microglia.

(A) Regulatory links predicted for *C1QA* in microglia. ATAC coverage tracks and corresponding expression levels in controls and FCD IIb tissues are depicted at the top. Regions highlighted in gray indicate opening peaks in FCD and red asterisks indicate Encode-annotated enhancers. The gene track shows the location of the *C1QA* locus in chromosome 1, and the bottom track shows all peaks contained in the chromosomal region.

(B) Regulatory links predicted for *C1QB* in microglia. Legends are defined as in (A) above.
