# Supplementary material for: Multimodal single-cell profiling reveals neuronal vulnerability and pathological cell states in focal cortical dysplasia
Source: iScience. 2024 Nov 6;27(12):111337. doi: 10.1016/j.isci.2024.111337 (PMC11617397; doi:10.1016/j.isci.2024.111337)
Supplement: Data S1. Institutional ethics committee approval [file mmc2.pdf]

Research Ethics Committee – Ethics Evaluation Report

RESEARCH PROJECT DATA

Research Title: BIOREPOSITORY
MOLECULAR GENETIC STUDIES IN NEUROPSYCHIATRIC DISEASES -PHASE I

Researcher: Iscia Lopes Cendes
Field: Human Genetic: Area 1
(This is a research involving Human Genetics not contemplated above);

Version: 2
CAAE: 12112913.3.0000.5404
Proponent Institution: University of Campinas Teaching Hospital (HC-Unicamp)
Main Sponsor: Own Financing

OPINION DATA

Report Number: 257.020
Report Date: 12/04/2013

Project Presentation:

This project refers to implantation of biorepository of neuro-psychiatric diseases and control cases. The study will predict the recruitment and gathering of biological material from 700 patients and 300 control subjects.
This project aims to study the molecular features of following diseases: epilepsy, malformations of cortical development, choreas, ataxias, paraparesis, dystonia, bipolar affective disorder, schizophrenia, muscular diseases, mitochondrial diseases, parkinson disease, stroke, dementias. The project is divided into sub-projects, with a detailed description of the strategies that will be used for each of these diseases.
Several molecular biology techniques will be used for the identification of known or new mutations, such as PCR, second and third generation sequencing, and bioinformatics analysis. In subproject 1 (epilepsy and malformations of cortical development), mutations will be evaluated through the implementation of sequencing technology, based on third generation equipment and a new electronic detection system, Ion Torrent® system.

In subproject 2, exome capture and sequencing will be performed on DNA samples from individuals affected by familial forms of juvenile myoclonic epilepsy (JME), temporal lobe epilepsy (TLE), benign rolandic epilepsy (BRE) and malformations of cortical development (MCD), which the mutations described were not identified. In this investigation, Illumina TruSeq® capture and enrichment kits will be used, as well as sequencing by Hi-Seq® 2000. After the sequencing, bioinformatics analyzes will be carried out in order to identify potentially pathogenic variants.
Patients will be recruited at outpatient clinics from HC-UNICAMP (Neurogenetics, Epilepsy, Epilepsy of Difficult Control, Children Epilepsy, Movement Disorders, Neuromuscular Diseases, Dystonia, Botulinum Toxin, Dementias, Neurovascular or Psychiatry). Peripheral blood, 20-30 mL, will be collected for the study, besides the clinical examination and anammese. Data from medical records will be collected.

Research Objective:
To identify mutations responsible for the respective phenotypes in a group of neuropsychiatric diseases, including: epilepsy, malformations of cortical development, choreas, ataxias, paraparesis, dystonia, bipolar affective disorder, schizophrenia, muscular diseases, mitochondrial diseases, parkinson disease, stroke, dementias.

Evaluation of Risks and Benefits:

The procedures to be performed are of small risks to the individual and the main of them is the blood collection by venipuncture.
As benefits, in some cases it will be possible to identify mutations associated with diseases in patients included in this study, which will imply the possibility of efficient algorithms for diagnosis and treatment. The authors caution that many mutations do not result in alterations of treatment, which is understandable when it comes to genetic studies.
The information generated during this project and which may have implications in the diagnostic confirmation of symptomatic individuals will be communicated to responsible for the follow-up of these patients in the sessions of orientation and genetic counseling of the Neurogenetic outpatient clinics, whose main researcher is responsible.

Research Comments and Considerations:

The project is well written and detailed, as well as the procedures that will be applied to controls volunteers and patients. The research project has scientific and social relevance at medium and long term. The study will be sponsored by owing researcher. The information on the site of collection and recruitment of the control group exams was added to the main project.

Considerations on the Obligatory Presentation Terms:

The Research consent form is well written; it is easily understood by the volunteer and provides the storage of biological material. All the terms of obligatory presentation provided by Resolution 196/96 and complementary, as well as the "Regulation of the Biorepository of Neuropsychiatric Diseases" are present.

Recommendations:

Nothing to declare

Conclusions or Pending and List of Inadequacies:

To the initial project, the information about the recruitment of volunteers from the control group was added, with initial prioritization for members of patient family, but no-disease carriers volunteers. The samples will be collected at the outpatient clinics of Genetics and Neurology HC-Unicamp and Hemocentro-Unicamp.

Status of Opinion:
Approved

Needs Assessment of CONEP:
No

Final Considerations at CEP's discretion:
Approved at the collegiate meeting on April 23, 2013

CAMPINAS, April 26, 2013

Signed by:

____
Fátima Aparecida Bottcher Luiz

(Coordinator)

Research Ethics Committee – Ethics Evaluation Report

RESEARCH PROJECT DATA

Research Title: BIOREPOSITORY
MOLECULAR GENETIC STUDIES IN NEUROPSYCHIATRIC DISEASES -PHASE I

Researcher: Iscia Lopes Cendes

Field: Human Genetic:
(This is a research involving Human Genetics – it does not need ethical analysis by the Central Ethics Committee- CONEP-);

Version: 4

CAAE: 12112913.3.0000.5404

Proponent Institution: University of Campinas Teaching Hospital (HC-Unicamp)

Main Sponsor: Own Financing

OPINION DATA

Report Number: 1.039.992

Report Date: 28/04/2015

Project Presentation:

This is an amendment to the original project, whose researcher requests authorization for data generated from the original project to be made available in public databases, assuring that no sample identifiers will be included.

As justification for the present request, the researcher comments that, with the advances of researches in the genomic field, nowadays, it is of great importance to share results of some molecular tests in public databases. In the sharing of genomic data, it is always insured that there will be no reference to sample identifiers, (identifier examples: name, filiation, address, hospital record number); however, relevant information for interpreting genomic data in the context of the researches, such as: sex, age, country and region of origin, diagnosis and other pertinent clinical information, will be included/shared.

In addition, the researcher requests exemption for reconsent for samples already stored under this biorepository. As a justification, the researcher comments the logistic difficulty in recontact the participants already included in the study.

Research Objective:

It shall be maintained in relation to the original project.

Evaluation of Risks and Benefits:

It shall be maintained in relation to the original project.

The participants already expressed their option in the original consent about their wishes to be contacted or not in case additional work were carried out with the same biological samples provided by them.

Thus, the researcher is committed to recontact individuals who wish so in order to obtain reconsent from those subjects, especially for those with regular visits to the university hospital. Only subjects who did not consent previously for new researches will be contacted for a new consent.

Research Comments and Considerations:

The amendment specifically requests:

1- Exemption from reconsent application in order to make data, generated in the original project, to be made public availability.

2- Application of reconsent for subjects who did not consent to new researches using the stored material. .

3- Application of an updated consent form to all new participants recruited, allowing he possibility of making the study data publicly available, provided that there will be no identification of the subject.

Considerations on the Obligatory Presentation Terms:

For this amendment, the following items were presented:

- letter of the principal researcher with presentation of the request with the respective justifications, in addition to the commitment to apply the new consent form for newly recruited subjects.

Recommendations:

Conclusions:

After considering the justification of the amendment andprevious odcuments presented, this Ethics Research Committee (CEP) decided the following:

1- **Approve** the newly presented (revised version) of the consent form presented by the researcher, which shall be used from this date to obtain consent from all newly recruited participants in this study.

2- **Approve** the application of the new consent form (reconsent), for individuals who wished to be recontacted in case of new research performed with samples previously donated and stored.

3- **Approve** exemption of application of new FCCT (reconsent), for participants who gave consent for new research and do not wish to be recontacted.

Endereço: Rua Tessália Vieira de Camargo, 126
Bairro: Barão Geraldo **CEP:** 13.083-887
UF: SP **Município:** CAMPINAS
Telefone: (19)3521-8936 **Fax:** (19)3521-7187 **E-mail:** cep@fcm.unicamp.br

4- The exemption of reconsent is **not approved** for participants who are being followed regularly at UNICAMP university hospital. In such cases, the newly approved consent form should be applied (reconsent).

Opinion Situation:

Approved.

CONEP appreciation:

It is not necessary.

Final Considerations at the Discretion of the CEP:

- The research subject must receive a copy of the consent term in full, signed by him.

- The research subject is free to refuse to participate or to withdraw their consent at any stage of the research, without any penalty and without prejudice to their care.

- The researcher must develop the research as outlined in the approved protocol. If the researcher considers discontinuation of the study, this must be justified and can only be performed after consideration of the reasons for discontinuation by the CEP, which approved it. The researcher must wait for the opinion of the CEP before discontinuation, unless when noticing risk or damage not anticipated by the participant or in case of finding the superiority of a diagnostic or therapeutic strategy offered to one of the research groups, that is, only if there is necessary immediate action to protect study participants.

- The CEP must be informed of any adverse effects or relevant facts that alter the normal course of the study. It is responsibility of the researcher to ensure immediate appropriate measures against serious adverse events occurred (even if it has been in another center) and send notification to the CEP and the National Health Surveillance Agency - ANVISA - along with its positioning.

- Any modifications or amendments to the protocol shall be submitted to the CEP clearly and succinctly, identifying the part of the protocol to be modified and their justifications. In the case of Group I or II projects previously submitted to ANVISA, the researcher or sponsor

Should also communicate immediately with this agency.

- Partial and final reports must be submitted to the CEP, initially six months after the date of this approval and at the end of the study.

CAMPINAS, April 28, 2015

Signed by:
Renata Maria dos Santos Celeghini
(Coordinator)

Endereço: Rua Tessália Vieira de Camargo, 126
Bairro: Barão Geraldo **CEP:** 13.083-887
UF: SP **Município:** CAMPINAS
Telefone: (19)3521-8936 **Fax:** (19)3521-7187 **E-mail:** cep@fcm.unicamp.br
